# Diagnostic accuracy and reliability of CT-based Node-RADS for colon cancer

**DOI:** 10.1007/s00261-024-04485-4

**Published:** 2024-07-08

**Authors:** Jakob Leonhardi, Matthias Mehdorn, Sigmar Stelzner, Uwe Scheuermann, Anne-Kathrin Höhn, Daniel Seehofer, Benedikt Schnarkowski, Timm Denecke, Hans-Jonas Meyer

**Affiliations:** 1 Department of Diagnostic and Interventional Radiology, University of Leipzig, Leipzig, Germany; 2 Department of Visceral and Transplantation Surgery, University Hospital Leipzig, University of Leipzig, Leipzig, Germany; 3 Department of Pathology, University Hospital Leipzig, University of Leipzig, Leipzig, Germany

**Keywords:** Computed tomography, Lymph node, Colon cancer

## Abstract

**Objective:**

The Node-RADS classification was recently published as a classification system to better characterize lymph nodes in oncological imaging. The present analysis investigated the diagnostic benefit of the Node-RADS classification of staging computed tomography (CT) images to categorize and stage lymph nodes in patients with colon cancer.

**Materials and methods:**

All patients were surgically resected and the lymph nodes were histopathological analyzed. All investigated lymph nodes were scored in accordance to the Node-RADS classification by two experienced radiologists. Interreader variability was assessed with Cohen’s kappa analysis, discrimination analysis was performed with Mann-Whitney-U test and diagnostic accuracy was assessed with receiver-operating characteristics (ROC) curve analysis.

**Results:**

Overall, 108 patients (*n* = 49 females, 45.3%) with a mean age of 70.08 ± 14.34 years were included. In discrimination analysis, the total Node-RADS score showed statistically significant differences between N- and N + stage (for reader 1: mean 1.89 ± 1.09 score for N- versus 2.93 ± 1.62 score for N+, for reader 2: 1.33 ± 0.48 score for N- versus 3.65 ± 0.94 score for N+, *p* = 0.001, respectively). ROC curve analysis for lymph node discrimination showed an area under the curve of 0.68. A threshold value of 2 resulted in a sensitivity of 0.62 and a specificity of 0.71.

**Conclusion:**

Node-RADS score derived from staging CT shows only limited diagnostic accuracy to correctly predict nodal positivity in colon cancer. The interreader variability seems to be high and should question the clinical translation for this tumour entity.

## Introduction

Colorectal cancer is the third most common tumour in men and the second in women, accounting for 10% of all tumour types worldwide [1–3]. Incidence is 25% higher in males and differs greatly between countries. With more than 600.000 deaths estimated each year, colorectal cancer is the fourth most common cancer-related cause of death globally [1–3]. Of the colorectal cancer, 1,096,000 new cases are colon cancers (CC) with distinctive differences to the cancers located of the rectum.

Approximately 75% of these patients will present with potentially curable disease and can be treated by surgical resection. Surgical treatment should include resection of the affected segment of bowel following the principles of complete mesocolic excision with resection of the embryologic package, including central vascular ligation of the supplying vessels and subsequent removal of the draining lymph nodes [1].

Lymph node staging is still of crucial importance for the prognosis and the therapy stratification in CC [4] and is usually performed by evaluation of the histopathological specimen. The occurrence of lymph node metastases is associated with an adverse clinical course with an indication for adjuvant chemotherapy [1]. In contrast, patients with stage I/II colon cancers show a considerable better outcome with a high rate of long-term survivors. The histopathological evaluation of at least 12 lymph nodes [1, 5] is recommended for reliable prediction of prognosis and deduction of the necessity of adjuvant chemotherapy.

Preoperative staging includes contrast enhanced computed tomography (CT) to rule out distant metastasis [4]. However, the diagnostic abilities of CT for local tumour (T) stage and nodal (N) stage in colon cancer is limited. A recent nation-wide study from the Netherlands demonstrated an overall radiological-pathological agreement for T stage of 59% and for N stage of 57%. The authors concluded that detection of lymph node metastases with CT remains unreliable. One can therefore deduce that new methods are needed to improve the diagnostic abilities of CT staging in CC.

Presumably, imaging markers could aid to overcome this current diagnostic weakness of CT, which was shown in different tumour entities [6–8]. A suggested method is Node-RADS (Node-Reporting and Data System), which was recently published as a promising classification system to standardize the categorization for lymph nodes in clinical staging [9]. It uses a 5-point probability scale ranging from 1 with very low likelihood for malignancy up to 5 with very high likelihood [9]. Yet, this classification system was only systematically investigated in few tumour entities, comprising gastric cancer, lung cancer, bladder cancer and perihilar cholangiocarcinoma [10–14]. One study has also evaluated the diagnostic abilities of Node-RADS in CC with promising results in a small patient sample with 67 CC [14]. However, more data regarding the diagnostic accuracy of Node-RADS is needed for the translation into clinical routine.

The purpose of the present study was to elucidate, whether CT-Node-RADS score can stratify locoregional lymph nodes and to improve the diagnostic performance for discrimination purposes in patients with CC.

## Methods

### Study design

This retrospective, observational study involving human participants was performed in accordance with the ethical standards of the institutional and/or national research committee and with the 1964 Helsinki declaration and its later amendments or comparable ethical standards. The study was approved by the local ethics committee (No. of the approval: 106-16-14032016).

The radiological database of one university hospital was retrospectively screened for patients with CC between January 2016 and December 2023.

Inclusion criteria consisted of available pre-surgical contrast enhanced CT images, histopathologically confirmed CC. All analyzed CT scans were acquired within one month before the surgical procedure. All patients underwent radical oncologic colon resection with curative intention with locoregional lymphadenectomy.

### Surgery and pathology

In all cases, the tumour resection was combined with a systematic lymph node dissection of the locoregional lymph nodes. As suggested by clinical guidelines, in every case at least 12 lymph nodes were examined [1, 5]. According to clinical routine all suspicious lymph nodes were removed and analyzed by histology. The lymph node stage was retrieved from the pathological report during clinical routine.

### Imaging technique

In all cases, CT was performed with a 128-slice CT scanner (Ingenuity 128, Philips, Hamburg, Germany) during clinical staging including the thorax, abdomen and pelvis. For every patient, intravenous administration of an iodine-based contrast medium (90 mL Imeron 400 MCT, Bracco Imaging Germany GmbH, Konstanz, Germany) was given at a rate of 2–4.0 mL/s via a peripheral venous line. All investigated CT images were obtained in portal venous phase after 70 s. Automatic bolus tracking was performed in the aorta descenders with a trigger of 100 Hounsfield units (HU). Typical imaging parameters were: 100 kVp; 125 mAs; slice thickness 1 mm; pitch 0.9.

### Lymph node score

The lymph nodes were scored according to the proposed Node-RADS classification [9]. Scoring was performed by two trained radiologists with 5 and 4 years of experience in oncological CT imaging analysis, respectively. Both readers were blinded to the pathological results and to each other’s results. For every patient the largest, most suspicious lymph node was scored.

**Fig. 1:**
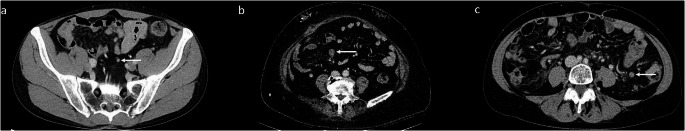
Representative cases of the patient sample. (**a**) 51-year old male patient, adenocarcinoma of the sigma, T3a, N0, M0, Node-RADS 1, short-axis-diameter 6 mm. (**b**) 78-year old female patient, adenocarcinoma of the distal colon transversum, T3b, N1b, M0, Node-RADS 5, short-axis-diameter 16 mm. (**c**) 50-year old female patient, adenocarcinoma of the colon descendens, T2, N1b, M0, Node-RADS 3, short-axis-diameter 9 mm

The scoring system ranges from 1 to 5 reflecting the level of probability of malignancy: “1—very low”; “2—low”; “3—equivocal”; “4—high”; “5—very high”. In the classification the two imaging features size and configuration are considered as the two main findings. The size was classified as enlarged, when short axis diameter was above 10 mm. Regarding configuration, the CT texture was assessed in a qualitative manner and categorized as homogenous, heterogeneous, focal or gross necrosis. The lymph node border can be described as smooth or irregular. Shape was defined as kidney bean with fat hilus or spherical without fat hilus. All features together resulted in the final lymph node category. Figure 1 displays 2 representative cases of the patient sample for illustration purposes.

### Statistical analysis

The statistical analysis and graphics creation were performed with SPSS (IBM, Version 25.0; Armonk, NY, USA). The collected data was first analyzed with descriptive statistics. Interreader variability was assessed with Cohen’s kappa analysis for the categorial parameters. Discrimination analysis was performed with Mann-Whitney test for continuous data and Fisher’s exact test for categorical data. Receiver-operating characteristics (ROC) curve analysis was used to test for diagnostic accuracy with area under the curve (AUC)-analysis. In all instances, two-sided p-*v*alues < 0.05 indicated statistical significance. A power calculation was performed with the assumption of an incidence of lymph node metastasis of 27% in a general population. The resulting power was calculated to be 81.7% for the present study.

## Results

Overall, 108 consecutive patients (*n* = 49 females, 45.3%) with a mean age of 70.1 ± 14.3 years were included in the present study.

Table 1 displays the demographics of the investigated patients. Figure 1 shows exemplary cases of the patient samples. Overall, 43 patients were nodal positive (38.9%), whereas 65 patients were nodal negative (61.1%). N1 stage was found in 21 cases (19.4%), N2 in 22 cases (20.4%).

**Table 1:** Demographic characteristics of the investigated patient sample

Parameter	N- (*n* = 65)	N+ (*n* = 43)	*p*-value
**Age (years)**	71.6 ± 13.2	67.7 ± 15.7	0.27
**Gender**			0.55
**(male, n, %)** **(female, n, %)**	34 (57.6) 31 (63.3)	25 (42.4) 18 (36.7)	
**pT**			**0.01**
1	3	1	0.54
2	14	2	**0.02**
3	40	24	0.56
4	8	16	**0.002**
**pM**			**0.001**
0	64	23	**0.001**
1	1	20	**0.001**
short axis diameter in mm	6.8 ± 3.33	10.7 ± 7.30	**0.001**

All tumours were adenocarcinomas. There were only 4 cases with a rare subtype, in two cases a mucinous adenocarcinoma and in two cases a signet-ring adenocarcinoma, respectively.

In 26 patients (24.1%) a metastasized stage was diagnosed with the following sites of distant metastasis, peritoneal in 14 cases (53.8%), liver in 13 cases (50.0%), lung in 4 cases (15.4%) and in case ovary (3.8%).

Among the metastasized patients 25 cases (96.1%) were nodal positive and one case was nodal negative.

When applying the commonly used threshold of 10 mm for short-axis-diameter to define malignant lymph nodes in clinical routine, 57/65 (87.7%) N- cases were correctly staged as N-, whereas 17/43 (39.5%) N + cases were correctly staged as N+. The resulting false positive rate is 8/65 (12.3%) and the false negative rate is 26/43 (60.4%).

When using a total Node-RADS-score of 4 or higher as threshold, 58/65 (89.2%) cases would have been correctly staged as N- and also 17/43 (39.5%) cases correctly as N+. The resulting false positive rate is 8/65 (12.3%) and the false negative rate is 26/43 (60.4%).

Using a fixed cut-off value for total Node-RADS-score of 3 and higher, an increase of correct staging for N + cases (24/43, 55.6%) can be accomplished, whereas over-staged cases inclined at almost the same rate (50/65, 77%). The resulting false positive rate is 15/65 (23.1%) and the false negative rate is 19/43 (44.1%).

### Discrimination analysis of Node-RADS score for N stage

The distribution of malignancy according to each Node-RADS score for both readers are shown in Table 2.

**Table 2:** Distribution of malignancy according to each Node-RADS score for both readers

Node-RADS score	Reader	Number of cases scored	Histopathologically confirmed malignancy
1	1	44	13 (29.5%)
	2	30	6 (20%)
2	1	25	6 (24%)
	2	22	11 (50%)
3	1	15	7 (46.7%)
	2	18	5 (27.8%)
4	1	10	5 (50%)
	2	17	6 (35.3%)
5	1	14	12 (85.7%)
	2	21	15 (71.4%)

Node-RADS-scoring resulted for reader 1 in a total of *n* = 44 for Node-RADS 1 (40.7%), *n* = 25 for Node-RADS 2 (23.1%), *n* = 15 for Node-RADS 3 (13.9%), *n* = 10 for Node-RADS 4 (9.3%) and *n* = 14 (13%) for Node-RADS 5.

For reader 2, the results were *n* = 18 for Node-RADS 1 (36%), *n* = 12 for Node-RADS 2 (24%), *n* = 6 for Node-RADS 3 (12%), *n* = 10 for Node-RADS 4 (20%) and *n* = 4 (8%) for Node-RADS 5.

Inter-reader agreement was only fair for the Node-RADS scoring (k = 0.35, *p* < 0.001).

For reader 1, Node-RADS 1 had a malignancy rate of 29.5% and for reader 2 0%, Node-RADS 2 had a malignancy rate of 24% for reader 1 and of 25% for reader 2, Node-RADS 3 had a malignancy rate of 46.7% for reader 1 and 75% for reader 2. Node-RADS 4 yielded a malignancy rate of 50% for reader 1 and 35.3% for reader 2, for Node-RADS 5 reader 1 reached a malignancy rate of 85.7% and reader 2 71.4%.

In discrimination analysis, the total Node-RADS score showed statistically significant differences between N- and N + stage (for reader 1: mean 1.89 ± 1.09 score for N- versus 2.93 ± 1.62 score for N+, for reader 2: 1.33 ± 0.48 score for N- versus 3.65 ± 0.94 score for N+, *p* = 0.001, respectively).

**Fig. 2:**
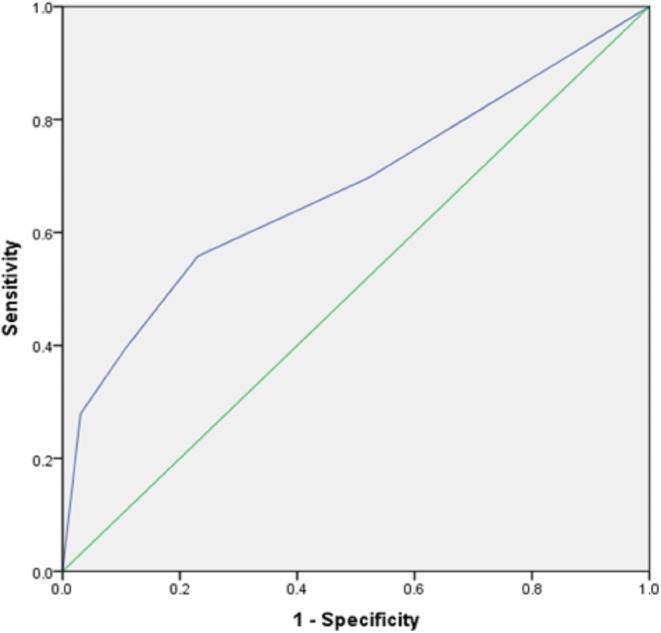
Results of the ROC curve analysis for discrimination of N- versus N + with total Node-RADS-Score, yielding an AUC of 0.68 [95 CI: 0.57–0.79]. A threshold value of 2 resulted in a sensitivity of 0.62 and a specificity of 0.71

ROC curve analysis for lymph node discrimination (N- versus N+) showed an area under the curve (AUC) of 0.68. A threshold value of 2 resulted in a sensitivity of 0.62 and a specificity of 0.71 (Fig. 2).

Short axis diameter reached statistically significant difference between N- and N + stage (mean 6.8 ± 3.3 mm for N- versus 10.7 ± 7.3 mm for N+, *p* = 0.001).

Node-RADS subcategory size also reached statistically significant difference between N- and N + stage (mean 1.11 ± 0.31 score for N- versus 1.42 ± 0.54 score for N+, *p* < 0.001).

Also, for both readers, the Node-RADS-subcategories shape (*p* = 0.002) and border (*p* = 0.002) achieved statistically significance with higher subcategory-scores correlating with higher likelihood of positive N-stages. However, the subcategory texture did not reach statistical significance (*p* = 0.12).

**Fig. 3:**
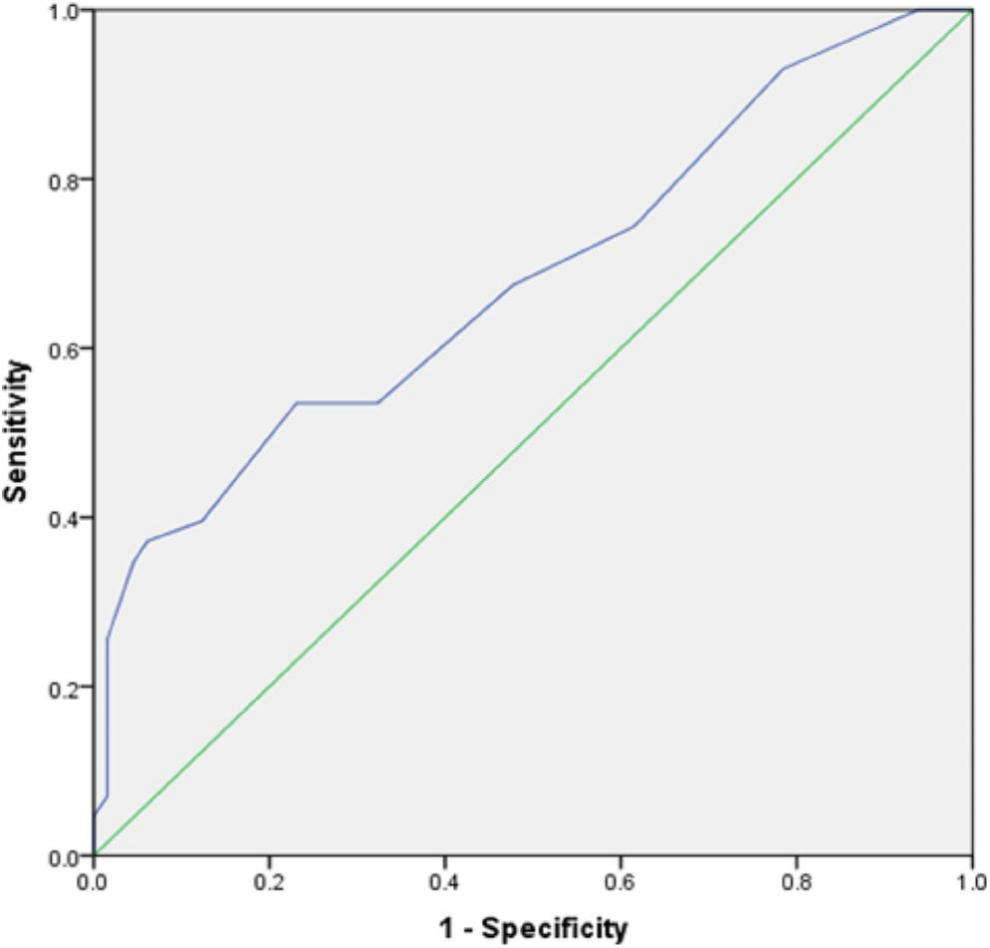
Results of the ROC curve analysis for discrimination of N- vs. N + with short axis diameter, the resulting area under the curve (AUC) is 0.68 [95% CI: 0.58–0.79]. Using a threshold value of 6.5 mm, sensitivity reached 0.67 and specificity 0.52

ROC curve analysis for lymph node discrimination (N- versus N+) using the short axis diameter resulted in an area under the curve of 0.68. Using a threshold value of 6.5 mm, sensitivity reached 0.67 and specificity 0.52 (Fig. 3).

No significant difference was found between the AUC for lymph nodes discrimination using total Node-RADS score and the AUC for discrimination with short axis diameter (*p* = 0.85).

Inter-reader agreement was fair for the subcategory shape (k = 0.24, *p* = 0.001), moderate for the subcategories border (k = 0.44, *p* < 0.001) and texture (k = 0.52, *p* < 0.001), but reached only slight values for the added-up total configuration score, with a Cohen’s kappa of k = 0.18 (*p* < 0.001).

## Discussion

The present analysis utilized the Node-RADS as a semiquantitative method to characterize lymph nodes in CC. As a key finding of the present study it can be concluded that Node-RADS has only a limited diagnostic accuracy to predict nodal metastasis.

Nodal status is of great prognostic relevance in patients with CC in an independent manner [1, 15]. It could alter preoperative surgical planning and could also improve clinical decision making regarding adjuvant chemotherapy indication before the final diagnosis [1]. Both aspects show that there is the crucial need for imaging modalities to improve the diagnostic accuracy for lymph nodes diagnosis in a non-invasive manner.

Conventional CT imaging has only a limited accuracy for nodal staging with an overall low reported accuracy [4, 16]. As demonstrated in a large recent study the sensitivity and specificity of CT to detect N1-N2 category were 62% and 70%, respectively [4].

To overcome these limitations of CT, several studies were undertaken to provide new findings to better discriminate nodal negative from nodal positive tumours. As reported by Hong et al. the largest short diameter of lymph node and presence of internal heterogeneity of lymph node showed significant association with malignant lymph node status (*P* < 0.001 and *P* = 0.041, respectively) [17]. The authors could report an AUC of 0.703 for the largest short diameter of lymph node (*P* < 0.001), and AUC of the presence of internal heterogeneity was 0.630 (*P* < 0.001) (17).

In another study by Cheng et al., a nomogram based on a triphasic contrast CT yielded an AUC of 0.71 (95% CI, 0.585–0.839) in the training cohort [18].

An interesting study employing deep learning achieved an AUC of 0.619 (95% CI 0.507–0.731) in the validation cohort and of 0.542 (95% CI 0.489–0.595) in the test cohort [19]. This demonstrated that the deep learning network was not superior than a conventional reading by the radiologist.

Mou et al. measured several diameters of the primary tumour and could show some association with a positive nodal stage [20]. This can be discussed as a surrogate of the T-stage, which is known to be an important risk factor for nodal positivity in higher stages [21]. As such, in T1 stage, there is 11.1% nodal positivity, in T2 stage 19.3% and in T3 stage (48.5%), as reported in a large Chinese cohort [21]. Beyond that, the number of metastatic lymph nodes may be influenced by the total number of lymph nodes examined [22], which is of clinical importance. The present study is based upon a representative university hospital with a high expertise in colorectal surgery.

An important field in current radiology is to standardize the radiological reporting. Node-RADS is a recently proposed scoring system to categorize several imaging findings of lymph nodes on cross sectional imaging and to aid to better report lymph node involvement in clinical routine [9]. There is no restriction of this classification regarding the localization of the lymph node or the oncological disease. In Node-RADS diameter of 10 mm is proposed as general threshold value in the short axis.

The merit of the present study is that it provides representative results for the novel Node-RADS classification in patients with CC. The diagnostic accuracy of the present study using Node RADS is lower compared to recent studies investigating lung cancer and gastric cancer.

A recent study from Italy could demonstrate higher diagnostic accuracy of the Node-RADS score in 67 patients with CC [14]. One could argue that these differences might be caused by different patient sample composition or center specific criteria. For gastric cancer, the sensitivity of Node-RADS 3 was 56.8% with a specificity of 90.7% and for Node-RADS 4 of 48.6% sensitivity and 98.1% specificity, respectively [13]. For both categories, the study could demonstrate a substantial interreader agreement (κ = 0.73 and 0.67, *p* < 0.01) [13]. Similar results were provided in lung cancer patients, which also showed a higher interreader agreement and better diagnostic accuracy [11].

However, the present study demonstrates a lower interreader agreement for the different categories of the Node-RADS classification. Especially shape and border showed weak and moderate agreement between the readers. One can assume that especially for CC patients with small lymph nodes below 10 mm in short axis diameter, the important characteristics for lymph node assessment according to the Node-RADS features is very reader dependent. This results in overall a weak interreader agreement of the score and of most clinical importance it results in only a similar diagnostic performance compared to the short-axis diameter.

It remains unclear, why the identified diagnostic accuracy for Node-RADS in CC seems to be rather limited. One could conclude that the categorization of the Node-RADS simply reflects the clinical reading of the radiologists, which is known to show a low diagnostic accuracy in clinical routine.

A clear threshold for malignancy cannot be provided by Node-RADS, which is also in good agreement with the previous analysis of this classification on mediastinal lymph nodes [11]. The malignancy rate for the Node-RADS group 5 was lower compared to previous studies [10, 11]. At best, Node-RADS 5 would be almost 100% nodal positivity.

Several limitations of the present analysis are to be addressed. First, it has a retrospective study design with possible known inherent bias. To reduce this possible bias the CT images were analyzed in a blinded manner to the clinical and pathological results. Second, the patient sample size is derived from one center, which could lead to a selection bias. Third, the experience of the readers for the current study is limited with 4 and 5 years, respectively. A more experienced radiologist might rate some cases in another manner. However, due to standardization, the Node-RADS score should be relatively reliable for different experience levels. Forth, there might be some matching bias between the presurgical CT images and the pathological results with the final nodal status. However, this cannot be adjusted for in a retrospective study design.

In conclusion, Node-RADS score derived from staging CT shows only limited diagnostic accuracy to correctly predict nodal positivity in colon cancer. The interreader variability seems to be high and should question the clinical translation for this tumour entity.

## Data Availability

The datasets of current study are available from the corresponding author on reasonable request.

## Abbreviations

AUC: Area under curve
CT: Computed tomography
CI: Confidence interval
CC: Colon cancer
Node-RADS: Node-Reporting and Data System
ROC: Receiver-operating characteristics
